# Toward an optimized assessment of adolescent psychopathology risk: Multilevel environmental profiles and child irritability as predictors

**DOI:** 10.1002/jcv2.12180

**Published:** 2023-06-13

**Authors:** Qiongru Yu, Brianna Hernandez, Conner Swineford, Nia Walker, Leigha MacNeill, Yudong Zhang, Lauren S. Wakschlag, Jillian L. Wiggins

**Affiliations:** ^1^ San Diego State University/University of California San Diego Joint Doctoral Program in Clinical Psychology San Diego California USA; ^2^ Department of Psychology San Diego State University San Diego California USA; ^3^ Department of Medical Social Sciences Feinberg School of Medicine Northwestern University Chicago Illinois USA; ^4^ Institute for Innovations in Developmental Sciences Northwestern University Chicago Illinois USA

**Keywords:** adolescence, longitudinal studies, risk factors

## Abstract

**Background:**

Adolescence is a developmental period during which youth experience vulnerability to psychopathology. To build the foundation for a parsimonious psychopathology risk calculator while capturing the complexity and dynamic nature of the environment, the current study aimed to identify distinct risk and resilience profiles with a wide range of environmental factors guided by Bronfenbrenner's biopsychosocial ecological system theory. The association between the early‐mid childhood risk profiles and psychopathology in adolescence were examined. Moreover, the predictive utility of early childhood irritability was evaluated in addition to the risk profiles.

**Methods:**

The data from Future of Families and Child Wellbeing Study a nation‐wide longitudinal study, were used in the latent profile analyses to identify the risk profiles with family, school, and neighborhood characteristics from 3 to 9 years old. To capture the socio‐environmental and cultural nuances, we extracted three subsamples, including Black/African American (*n* = 2587), Hispanic/Latinx (*n* = 1577), and White (*n* = 776) for separate analyses. Risk profile memberships were used to predict adolescence psychopathology, including depression, anxiety, attention deficits, oppositional defiant disorder, and conduct disorder symptoms. The predictive utility of early childhood irritability above and beyond environmental risk profiles was evaluated using stepwise regression.

**Results:**

Three risk profiles were identified in the Hispanic/Latinx and Black/African American subsamples, while four profiles were identified in White subsample. Almost all risk profile membership predicted both internalizing and externalizing psychopathology, while some profiles are predictive of externalizing symptoms only. Higher level of irritability predicted higher symptomatology in all five mental health outcomes above and beyond the environmental profiles.

**Conclusions:**

Distinct risk and resilience profiles primarily driven by parent and family characteristics were identified for all three major race/ethnicity groups. Our findings lay the foundation for a more efficient multi‐tiered information gathering process in mental health clinical settings to aid the decision making for intervention and prevention.

**Key points**

- Distinct risk and resilience profiles primarily driven by parent and family characteristics were identified for all three major race/ethnicity groups.
- Risk and resilience profile membership in all race/ethnicity groups are predictive of psychopathology in adolescence.
- Early childhood irritability is predictive of adolescence psychopathology above and beyond environmental risk and resilience profiles.
- The findings can inform development of strategic assessment planning tools that prioritize information gathering in certain domains and including irritability as an efficient and robust risk indicator.

## INTRODUCTION

Adolescence is a key turning point in development when a confluence of factors (neurobiological maturation, social and academic transitions, normative and non‐normative stressors) creates a “perfect storm” of vulnerability to psychopathology (McLaughlin et al., 2015). Indeed, many forms of internalizing and externalizing psychopathology onset or increase in adolescence (Merikangas et al., 2010), setting youth up for trajectories toward entrenched psychiatric disorders and detrimental outcomes in adulthood (Terhi Aalto‐Setälä et al., 2002). Being able to predict risk for adolescent internalizing and externalizing symptoms by adolescence is crucial to head off downstream negative effects in adulthood (Mittal & Wakschlag, 2017). Prior research has identified a wide array of environmental risk and resilience factors, and burgeoning research holds the promise of combining such factors to generate a probabilistic risk score (MacNeill et al., 2021); yet practically speaking, clinicians “in the trenches” rarely have the resources to assess the entire complement. Indeed, an information gathering approach that draw data from multiple levels is critical for translating the vast body of research to efficient clinical decision making. As a step toward this end, we demonstrate the utility of such approach through a subgrouping strategy, whereby we condense the vast potential variation in risk/resilience information into a few common profiles of youth who are characterized by a common pattern of risk and resilience factors and share a pathway toward outcomes. Thus, our long‐term goal is to build a foundation for a multi‐tiered information gathering approach that integrates information from across levels to derive profiles of children, which may reduce decisional uncertainty in recommending interventions for children of different risk profiles.

Race, which may involve different social experience, resources, discrimination, geographical locations, and many other unmeasured environmental factors, is associated with experiences of risk and resilience factors (Bailey et al., 2017; Harnett, 2020). Similar experiences may have different meanings and impact on individuals from different cultures, and these nuances may provide important contexts for understanding of risk and resilience profiles in underrepresented groups in research (Arrington & Wilson, 2000). In this study, we leverage a longitudinal, nationwide sample sufficiently powered to derive risk and resilience profiles in early‐middle childhood within each of three major racial/ethnic groups (Black/African American, Latinx/o/a/Hispanic, white non‐Hispanic). We investigate within‐group variation in each of these racial/ethnic groups in order to address the specific nuances and pressures that are often overlooked in research that combines all participants, and to avoid group‐wise comparisons that can perpetuate deficit models (Persell, 1981; Wright et al., 2022). We also examine the degree to which prediction based on environmental‐level factors may be improved by the addition of brief, child‐level assessment, focusing on early irritability symptoms given their demonstrated transdiagnostic predictive utility (Wiggins et al., under review).

### Holistic and multilevel approach to environmental factors

The complexity of multiple levels of environmental factors and the dynamic changes during the developmental period from early to mid‐childhood, a formative time in which children begin to interact more actively with their school and communities (Mah & Ford‐Jones, 2012), pose challenges in forming a prediction model that captures the variation of different environmental factors with which the children interact. Bronfenbrenner's biopsychosocial ecological system proposes that child development happens within several interconnected environmental systems that capture the context of family, peer, school, communities, and the chronicity and change of contexts (Bronfenbrenner, 1977; Paat, 2013). Previous studies have identified a large set of candidate environmental risk factors across these levels that are associated with adolescent mental health. Family environment, including parental psychopathology, child abuse, and neglect is linked to internalizing and externalizing symptoms in adolescence (Burstein et al., 2010; Dunn et al., 2011; Heleniak et al., 2016; Moylan et al., 2010). In particular, the association between child psychopathology and maternal depression has been widely studied and extensively documented (e.g., Kuckertz et al., 2018; Wiggins et al., 2014). Outside of the home environment, once children enter school age, they spend a large amount of time at school, and interactions with peers and teachers are important contributors to their mental well‐being. Indeed, better school connectedness is a potential resilience resource, as it has been found to be protective for depressive and anxiety symptoms in adolescents in a community prospective study (Shochet et al., 2006). Moreover, peer victimization is linked to a wide range of mental health problems, increased self‐harm and suicidal thoughts, and has an impact on adolescents' brain development in regions that are implicated in the development of generalized anxiety disorders (Quinlan et al., 2020; Reijntjes et al., 2010; Takizawa et al., 2014). The neighborhood or community, the greater environment that surrounds adolescents' households, can be a source of material or emotional support, serving as a resilience factor, or conversely a source of risk via instability and exposure to violence (Copeland‐Linder et al., 2010). These factors also vary depending on developmental timing across the early to mid‐childhood period. For example, maternal depression earlier (child ages 1–3 years) as compared to later (ages 5–9) in development was found to have a greater impact on child psychopathology symptoms (Wiggins et al., 2014) whereas harsh parenting later on (ages 5–9) was more impactful than earlier in development (ages 1–3) (Wiggins et al., 2015).

Whereas the research that identified each of these candidate environmental risk/resilience factors is crucial to understand the separate contributions, examining the combination of environmental factors holistically is particularly important as well, as the multiple levels of factors may have more complex synergistic or antagonistic versus simple additive effects on mental health. Moreover, despite that a large complement of factors all contribute to the mental health outcomes to a varying degree, identifying the major underlying patterns and understanding such patterns' associations with psychopathology can inform clinical decision making in prevention and intervention.

Indeed, clinicians often work under the circumstances in which they need to make clinical decisions without having every piece of information of the child. To improve the practical clinical utility of prediction research, translation must pragmatically consider how to make accurate assessments of risk on “imperfect,” incomplete information. One potential approach is to reduce the potential patterns of risk/resilience factors by identifying subgroups of individuals who have similar patterns, that is, through latent profile analysis. In particular, Zeiders et al. (2013) has used this method to examine the risk profiles in Mexican American youth in 5th grade using variables selected under the ecological framework and described the association between profiles and mental health outcomes. Zeiders et al. (2013) identified three quantitatively and qualitatively different risk profiles: low risk, moderate risk‐language (i.e., language hassles), and high risk‐peer (i.e., association with deviant peers, peer conflict, and peer ethnic discrimination) groups in Mexican American adolescents and found that the high risk‐peer group displayed significantly higher level of mental health symptomatology as compared to low risk and moderate risk‐language group.

### Child characteristics

In addition to environmental contexts, elevated early childhood irritability, defined as increased proneness to dysregulated anger relative to peers, is a transdiagnostic neurodevelopmental vulnerability marker (Wakschlag et al., 2018). Elevated irritability in early childhood (age 3 up to 5 years) has been found to be a robust predictor for both internalizing and externalizing symptoms at the transition to adolescence (Wiggins et al., 2018). Early childhood irritability is actionable for psychopathology screening given recent measurement developments that facilitate its pragmatic, wide‐spread implementation (Wakschlag, Davis, et al., under review; Wiggins et al., 2018). However, when measured alongside environmental attributes, the additive predictive value of this transdiagnostic predictor has yet to be evaluated. Combining the most promising child and environmental markers for risk will be crucial to optimize prediction for future translation to a risk calculator.

### Current study

Thus, to fill the gaps in the literature, the current study seeks to identify the risk and resilience profiles among children from three major racial/ethnic groups and examine how the profiles, along with early childhood irritability, can be utilized to identify children at risk for adolescent psychopathology. To accomplish this, first, we derive profiles of environmental risk/resilience patterns across early‐middle childhood (ages 3–9 years); second, we examine predictive validity of early‐middle childhood environmental risk/resilience profiles for mental health outcomes in adolescence (age 15); third, we quantify the extent to which early childhood irritability (age 3) improves the prediction of adolescent mental health outcomes above and beyond environmental risk/resilience profiles. We expect that we will identify within racial/ethnic group profiles that represent both normative levels of risk and resilience factors, as well as elevated patterns of such risk and/or resilience factors.

## METHODS

### Participants

Data were from the Future of Families and Child Wellbeing (FFCW) study, an on‐going longitudinal study that follows a cohort of 4898 children born in 20 major U.S. cities between 1998 and 2000 (Reichman et al., 2001), with time points at birth, ages 1, 3, 5, 9, and 15 years. The benefit of this population‐based sample is that groups that are often missed in other studies are represented. Moreover, it is enriched for risk, so we can detect patterns that might otherwise be obscured. The analytic sample comprised three subsamples: *n* = 1577 children whose father, mother, or both identified as Hispanic; *n* = 2587 children whose father, mother, or both identified as Black/African American; and *n* = 776 children whose father and mother both identified as White (Table 1). Multiracial individuals who have a Black/African American parent and a Hispanic parent were included in both Black/African American and Hispanic subsamples, and those who have a White parent and a Black/African American or Hispanic parent were classified in the Black/African American or Hispanic group. Retention varied across measures and timepoints (45.9%–99.8%, see Supporting Information S1 for details on each measure), and all available data were used; analyses (see below) leveraged full information maximum likelihood (McCartney et al., 2006), which is highly robust to data missing at random.

**TABLE 1:** (A–C) Demographics information of the Hispanic/Latinx, Black/African American, and White subsamples.

Hispanic	Overall (*n* = 1577)	HL‐first generation immigrant/low harsh parenting and neglect (*n* = 1226)	HL‐nonimmigrant/high harsh parenting/high bullying (*n* = 274)	HL‐school violence control/moderate harsh parenting (*n* = 77)
Predictive characteristics		Normative gray line	Externalizing orange line	Broad‐spectrum blue line
Male, *n* (%)	810 (51.4%)	613 (50%)	152 (55.5%)	45 (58.4%)
Immigration status, *n* (%)				
Nonimmigrant	398 (36.1%)	268 (32.2%)	104 (50.2%)	26 (41.2%)
First‐generation immigrant^a^	545 (49.4%)	454 (54.5%)	65 (31.4%)	26 (41.3%)
Second‐generation immigrant^b^	160 (14.5%)	111 (13.3%)	38 (18.4%)	11 (17.5%)
Maternal education, *n* (%)				
Less than high school	757 (48.1%)	610 (49.8%)	110 (40.3%)	37 (48.1%)
High school or equivalent	424 (26.9%)	328 (26.8%)	74 (27.1%)	22 (28.6%)
Some college or tech school	325 (20.6%)	229 (18.7%)	83 (30.4%)	13 (16.9%)
College or graduate school	69 (4.4%)	58 (4.7%)	6 (2.2%)	5 (6.9%)

Black/African American	Overall (*n* = 2587)	BAA‐low harsh parenting and neglect/low maternal depression (*n* = 1713)	BAA‐high harsh parenting/high neglect (*n* = 611)	BAA‐moderate harsh parenting/high neglect/chronic maternal depression (*n* = 263)
Predictive characteristics		Normative gray line	Externalizing blue line	Broad‐spectrum orange line
Male, *n* (%)	1358 (52.5%)	853 (49.8%)	365 (59.7%)	140 (53.2%)
Immigration status, *n* (%)				
Nonimmigrant	1252 (81.6%)	773 (79.1%)	335 (84.3)	144 (89.9%)
First‐generation immigrant^a^	156 (10.2%)	122 (12.5%)	28 (7.1%)	6 (3.8%)
Second‐generation immigrant^b^	126 (8.2%)	82 (8.4%)	34 (8.6%)	10 (6.3%)
Maternal education, *n* (%)				
Less than high school	867 (33.6%)	575 (33.6%)	200 (32.7%)	92 (35.1%)
High school or equivalent	911 (35.3%)	581 (34.0%)	218 (35.7%)	112 (42.7%)
Some college or tech school	664 (25.7%)	400 (25.7%)	172 (28.2%)	52 (19.8%)
College or graduate school	141 (5.5%)	114 (6.7%)	21 (3.4%)	6 (2.3%)

White	Overall (*n* = 776)	W‐first generation immigrant/low harsh parenting and neglect (*n* = 410)	W‐low maternal education/high neighborhood safety (*n* = 66)	W‐high neglect/moderate harsh parenting (*n* = 136)	W‐high harsh parenting/high peer bullying (*n* = 164)
Predictive characteristics		Normative gray line	Externalizing blue line	Broad‐spectrum/CD orange line	Broad‐spectrum/ODD yellow line
Male, *n* (%)	407 (52.4%)	209 (51.0%)	26 (39.4%)	75 (55.1%)	97 (59.1%)
Immigration status, *n* (%)					
Nonimmigrant	508 (91.4%)	251 (80.7%)	43 (89.6%)	59 (82.6%)	119 (88.2%)
First‐generation immigrant^a^	50 (8.2%)	39 (12.5%)	0 (0%)	6 (5.2%)	5 (3.7%)
Second‐generation immigrant^b^	51 (8.4%)	21 (6.8%)	5 (10.4%)	14 (12.2%)	11 (8.1%)
Maternal education, *n* (%)					
Less than high school	112 (14.4%)	37 (9.0%)	21 (31.8%)	21 (15.4%)	33 (21.0%)
High school or equivalent	185 (23.8%)	85 (20.7%)	30 (45.5%)	19 (14.0%)	51 (31.1%)
Some college or tech school	212 (27.3%)	110 (26.8%)	11 (16.7%)	40 (29.4%)	51 (31.1%)
College or graduate school	267 (34.4%)	178 (43.4%)	4 (6.1%)	56 (41.2%)	29 (17.7%)

^a^ Indicates at least one foreign‐born parent

^b^ Indicates at least one foreign‐born grandparent but U.S.‐born parents.

### Measures

The variables used in the current study were collected through child‐, mother/primary caregiver‐, and teacher‐report questionnaires administered through phone or home/school visits.

#### Environmental measurements

##### Family characteristics

*Immigration status* was dummy coded into first‐generation immigrant and second‐generation immigrant, with non‐immigrant as the reference category.

*Harsh and neglectful parenting* was assessed by Parent‐Child Conflict Tactic Scale (CTSPC) via mother/primary caregiver report at child ages 3, 5, and 9. The physical assault subscale assesses the frequency of behaviors such as spanking child on the bottom with their bare hand, pinching the child, or slapping child on the hand, arm, or legs. The psychological abuse subscale assesses the frequency of behaviors such as shouting, screaming, or yelling at child, threatening to spank or hit, and swearing or cursing at child. Neglect assesses behaviors such as parent being so caught up with their own problems that they were not able to show or tell their child that they loved him/her, having to leave the child at home even when they thought some adult should be with the child, and not able to make sure the child has food or medical care when needed. Subscale scores for physical assault and psychological abuse were computed by averaging the items for each time point, thus resulting in two mean scores that indicate a range of harsh parenting behaviors that may include abuse. The internal consistency (Cronbach's alpha) is 0.61 0.60, and 0.70 for physical assault respectively for age 3, 5, and 9, and 0.56, 0.61, and 0.70 for psychological abuse. To mitigate variation in reliability and to decrease model complexity while highlighting the longitudinal nature of the data, latent intercepts and slopes were estimated for physical assault and psychological abuse using latent growth analyses and were then used as indicators in the profile analysis. The linear growth curve models with slope and intercept demonstrated good model fit (physical assault: comparative fit index [CFI] = 0.995, root mean square error of approximation [RMSEA] = 0.034; psychological abuse: CFI = 0.999, RMSEA = 0.020). The intercept for the physical assault and psychological abuse models were extracted to represent the early physical assault and psychological abuse, and the slopes were extracted and conceptualized as the change over time. The linear growth curve model for the neglect subscale score failed to converge, which may be due to very few parents endorsing neglect items. Therefore, to capture chronicity, neglect was recoded as “0” for never endorsing any neglect items, “1” for endorsing at least one neglect item at one time point, and “2” for endorsing at least one neglect item at multiple time points.

*Maternal depression* was measured at child's ages of 1, 3, 5, and 9 using the Composite International Diagnostic Interview Short Form (Green et al., 2010). Maternal depression in child's life was calculated as never depressed, depressed once, and chronically depressed (2 or more times), based on the number of time points at which the mother met the criteria for depression then dummy coded into “maternal depression (once)” and “maternal depression (chronic),” with “never depressed” as the reference category.

*Maternal education* was reported by the mother at baseline with four categories “less than high school,” “high school or equivalent,” “some college,” and “college or graduate school.” Maternal education was included in the model as a proxy for family's socio‐economic status.

*Parent cultural identity* was reported by father and mother using two 4‐point Likert‐type items: “I feel an attachment to my own race/ethnic heritage” and “I participate in cultural practices of my own group.” The measure has been used in previous studies (Lazarevic et al., 2020), and the mean score of the items reported by mother and father were calculated to represent parent cultural identity with higher scores indicating stronger cultural affiliation.

##### Neighborhood characteristics

*Neighborhood cohesion* at age 9 was measured by the five‐item Social Cohesion and Trust Scale (Sampson et al., 1997), which assessed the level of collective efficacy and trust. Mother responded to the questions regarding neighborhood cohesion (e.g., “this is a close‐knit neighborhood”) with 4‐point Likert scale from “strongly agree” to “strongly disagree” (Cronbach's alpha = 0.87). All items were reverse coded to compute an average score with higher score indicating higher level of cohesion.

*Neighborhood safety* was measured by four teacher‐reported items targeting criminal activity, gun violence, selling and using of drugs, and litter or broken glass in the neighborhood surrounding the school, using a three‐point scale from 1 = a big problem, 2 = somewhat of a problem, and 3 = no problem (Cronbach's alpha = 0.93), resulting in an average score with higher score indicating better perceived neighborhood safety.

##### School characteristics

*Peer bullying* at age 9 was measured by the child's report on the frequency of bullying, including “pick on you,” “hit you,” “take away things,” and “left out of activities.” Child responded to interviewer's question items using responses ranging from 0 = not once in the past month, 1 = 1–2 times in the past month, 2 = once a week, 3 = several times per week, and 4 = every day (Cronbach's alpha = 0.67). An average score across the items were calculated, with higher score indicating more frequent bullying victimization from peers.

*Classroom climate* was measured by six teacher‐reported items, for example, “Routine administrative duties and paperwork interfere with my teaching.” Some items were reverse coded in a way that higher score indicates more positive attitude. The Cronbach's alpha is 0.71.

*School violence control measures* were assessed by nine binary teacher‐reported items, which asked if the school implemented procedures to curb violence at school, such as the use of metal detectors and security guards. Higher number indicates application of more violence control measures.

*School connectedness* at age 9 was assessed by four child‐reported items that assess the degree of inclusiveness, closeness, happiness, and safety the child experiences at school, for example, “how often did you feel like you were part of your school?” (Cronbach's alpha = 0.70) Higher score indicates stronger connectedness to school.

#### Child trait predictor

Child's irritability at age 3 was computed using the items from Child Behavior Checklist (Achenbach, 1992), including “temper tantrums or hot temper,” “stubborn, sullen or irritable,” “sudden changes in mood or feelings,” and “easily frustrated,” as in prior work (Wiggins et al., 2014). Mothers rated the items with 0 = not true, 1 = somewhat or sometimes true, and 2 = very true or often true. An irritability score was calculated as the mean score of the four items, which have been shown to comprise a single factor (Wiggins et al., 2014).

#### Mental health outcomes

The mental health outcomes of interest at age 15 were assessed with the Child Behavior Checklist (Achenbach & Rescorla, 2001), a parent‐report questionnaire. Severity of depression, anxiety, attention deficits, oppositional defiant problems, and conduct problems were measured using DSM‐oriented subscales, to facilitate comparisons to clinical diagnoses while preserving dimensionality. The scales demonstrated acceptable to good internal consistency with Cronbach's alpha of 0.70, 0.67, 0.78, 0.81, and 0.81 respectively.

### Analytic plan

#### Aim 1: Derive profiles of environmental risk/resilience patterns across early‐middle childhood (ages 3–9 years)

Latent profile analyses were used to identify distinct subgroups of children based on their risk and resilience factors. This person‐centered modeling approach allows for the identification of subgroups within the sample based on a set of variables. The risk and resilience characteristic indicators in raw score scales, including family, school, and neighborhood characteristics, were entered into the latent profile analysis models. Latent profile models with 1–6 classes were estimated in Mplus version 8 for Hispanic/Latinx, Black/African American, and White groups separately. We decided to conduct separate analyses for each racial/ethnic group rather than develop the profiles across the entire sample and test for invariance. We chose the former approach for two reasons: first, our goal was to describe within‐group variation, as recommended for racial/ethnic research (Celious & Oyserman, 2001; Whitfield et al., 2008); second, pooling data across groups to test a model for invariance would involve contrasting the groups with a “norm” based on the majority and may inadvertently perpetuate the idea that Whiteness is a norm. The number of classes was determined by a comprehensive consideration of Vuong‐Lo‐Mendell‐Rubin Likelihood Ratio Test (VLMR‐LRT), Akaike Information Criterion (AIC), Bayesian Information Criterion (BIC), sample size‐adjusted BIC (sBIC), entropy, and interpretability. Smaller AIC and BIC and entropy closer to 1 indicated better model fit, and a significant VLMR‐LRT indicates the utility of an additional class. Because subsequent analyses with class membership as a predictor variable were planned, the size and proportion of the smallest class were also taken into consideration when selecting the number of classes to avoid estimation error due to small group size (minimum 2% of total). To preserve the power to detect racial/ethnic variation within the racial/ethnic groups, we leveraged the full sample for profile identification. Future studies may wish to focus on replicating the profiles in independent samples.

#### Aim 2: Examine predictive validity of early‐middle childhood environmental risk/resilience profiles for mental health outcomes in adolescence (age 15)

After determining the optimal number of classes, group differences in mental health outcomes between the latent classes at age 15 were examined. Most likely class membership was used to predict mental health outcomes (each outcome separately) in a series of regression models, corrected for multiple comparison false discovery rate. In addition, the Bolck‐Croon‐Hagenaars (BCH) method was performed as a supplementary analysis to account for potential uncertainty in latent class membership estimation (Asparouhov & Muthén, 2014).

#### Aim 3: Quantify the extent to which early childhood irritability (age 3) improves the prediction of adolescent mental health outcomes above and beyond environmental risk/resilience profiles

Stepwise regression analyses with each DSM‐oriented subscale scores as outcome variables were performed, with most likely class membership entered into the model as the sole predictor for the psychopathology symptoms, followed by the irritability score at age 3. The change in model fit after adding each variable was evaluated by examining the variance explained.

## RESULTS

### Aim 1: Derive profiles of environmental risk/resilience patterns across early‐middle childhood (ages 3–9 years)

The model fit indices for Hispanic/Latinx, Black/African American, and White subsamples are presented in Table 2. The parameter estimates for the selected models are presented in Supporting Information S1: Table  S1–S3.

**TABLE 2:** Latent profile analysis model fit indices.

	AIC	BIC	Entropy	VLMR test *p* value
Hispanic				
1 profile	21,912.878	22,073.777	N/A	N/A
2 profiles	20,973.225	21,230.662	0.704	0.0000
**3 profiles**	**20,609.598**	**20,963.575**	**0.795**	**0.0291**
4 profiles	20,178.458	20,628.973	0.732	0.0103
5 profiles	19,944.658	20,491.712	0.727	0.1289
6 profiles	19,758.250	20,401.844	0.750	0.0941
Black/African American				
1 profile	40,080.977	40,256.725	N/A	N/A
2 profiles	38,603.755	38,884.951	0.662	0.0000
**3 profiles**	**37,970.153**	**38,356.798**	**0.713**	**0.0092**
4 profiles	37,516.507	38,008.600	0.759	0.0595
5 profiles	33,201.687	33,799.229	0.749	0.0145
6 profiles	32,673.553	33,376.543	0.803	0.0053
White				
1 profile	9993.661	10,133.286	N/A	N/A
2 profiles	9216.556	9439.955	0.912	0.0207
3 profiles	8918.042	9225.216	0.747	0.0003
**4 profiles**	**8723.701**	**9114.649**	**0.747**	**0.0386**
5 profiles	8610.577	9085.300	0.752	0.5969
6 profiles	8510.015	9068.513	0.755	0.2949

*Note*: The selected models are bolded.

Abbreviations: AIC, Akaike information criterion; BIC, Bayesian information criterion; VLMR, Vuong–Lo–Mendell–Rubin likelihood ratio test.

#### Hispanic/Latinx subsample

Of models estimated with 1‐ to 6‐class solutions, the 3‐class solution was identified as the best fitting model based on comprehensive consideration of model fit indices and interpretability for the Hispanic/Latinx group. Among the latent profile models with the Hispanic/Latinx sample, the 3‐class solution had the highest entropy (0.795) and a significant VLMR‐LRT (*p* < 0.029), indicating superiority over a 2‐class model. Another potential candidate was the 4‐class solution, which had lower AIC, BIC, and sBIC compared to the 3‐class solution. It also had a significant VLMR‐LRT but a lower entropy of 0.732. Upon examination of the conditional response means and probabilities, however, the additional class in the 4‐ versus 3‐class model was comprised by splitting the three classes into finer groups of varying levels, which did not add substantial information for the characterization of subgroups. Therefore, we selected the 3‐class model for the Hispanic/Latinx subsample based on the evidence of both good model fit and parsimony.

Considering the conditional response means and probabilities (Figure 1A), the Hispanic/Latinx (HL)‐first generation immigrant/low harsh parenting and neglect group (gray line, *n* = 1226, 77.7%) was characterized by a high probability of being a first‐generation immigrant and less negative parent‐child interaction, that is, lowest neglect, psychological abuse, and physical assault. The HL‐nonimmigrant/high harsh parenting/high bullying group (orange line, *n* = 274, 17.4%) was characterized primarily by nonimmigrant status and more negative parent‐child interaction (i.e., physical assault and psychological abuse) and elevated peer bullying as compared to the HL‐first generation immigrant/low harsh parenting and neglect. The HL‐school violence control/moderate harsh parenting group (blue line, *n* = 77, 4.88%) was characterized by moderately negative parent‐child interactions (i.e., early physical assault and psychological abuse) and higher school violence control.

FIGURE 1(A) Latent risk profiles for the Hispanic/Latinx subsample. (B) Latent risk profiles for the Black/African American subsample. (C) Latent risk profiles for the White subsample.

**Figure:**
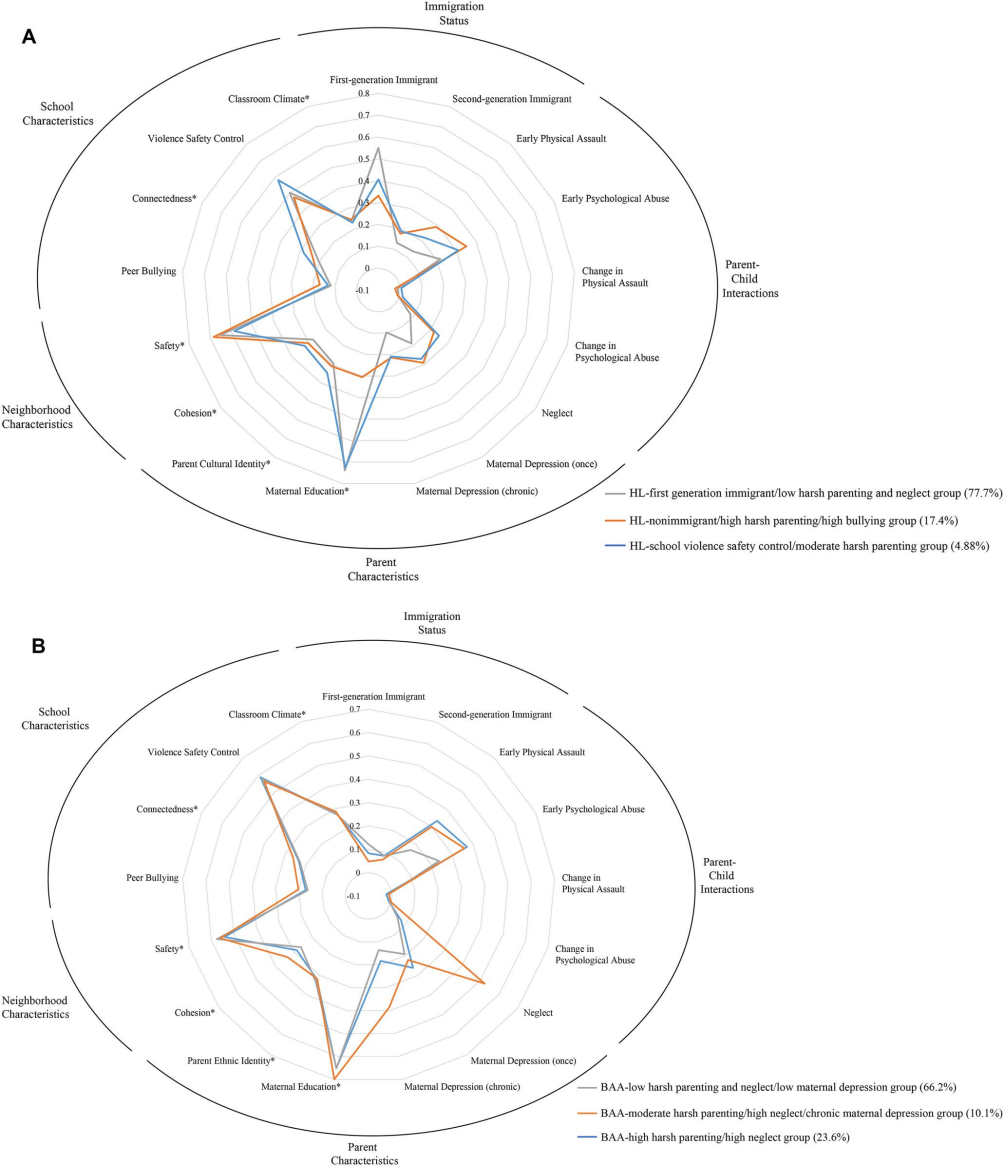

**Figure:**
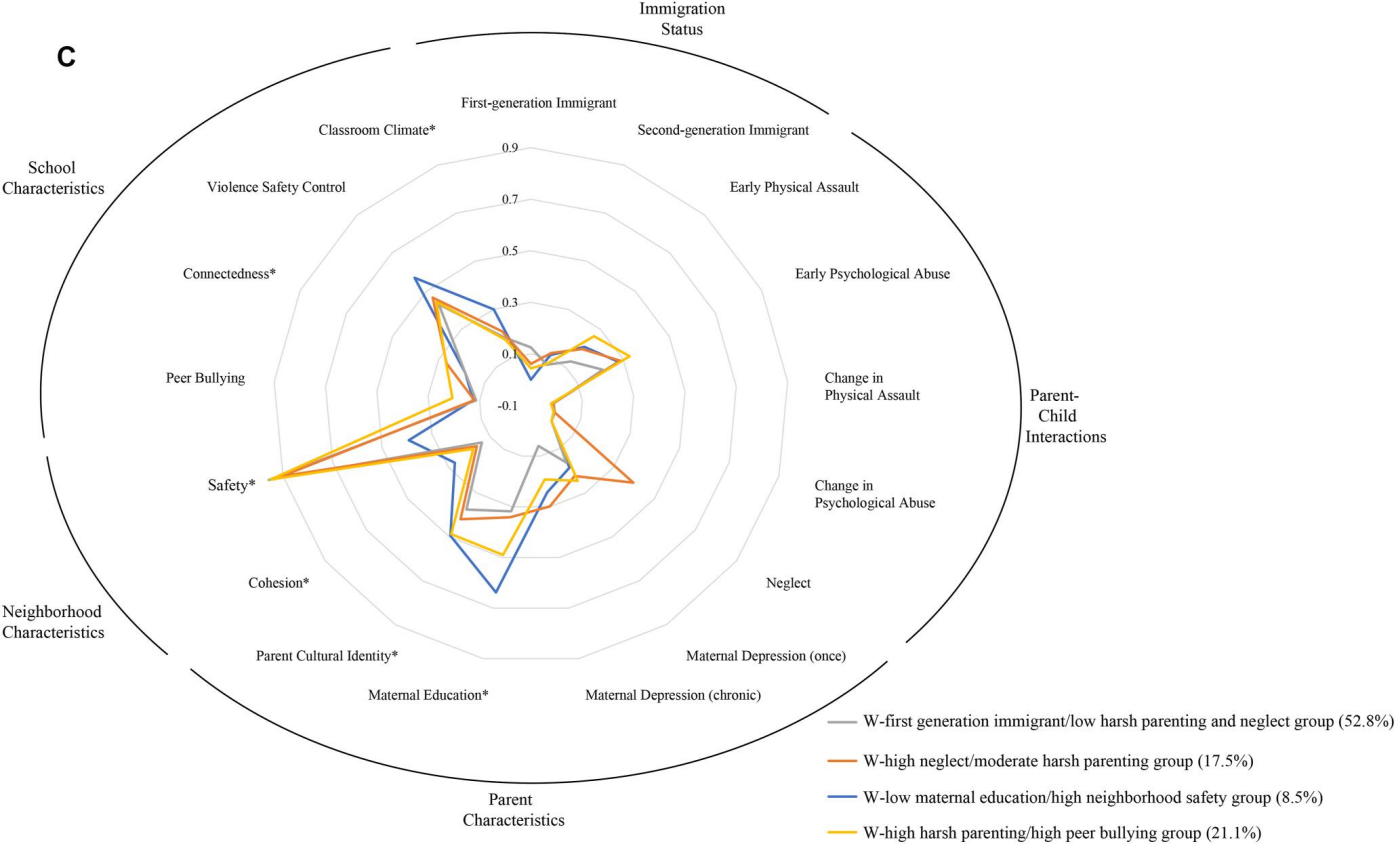

#### Black/African American subsample

Similarly, for the Black/African American subsample, the 3‐class solution was selected to be the final model as it demonstrated the best balance between model fit, parsimony, and interpretability. The 3‐class model had a significant VLMR‐LRT (*p* = .009) while the VLMR‐LRT for the 4‐class model was not significant (*p* = .06), which indicated that 3 classes were sufficient. The 3‐class model also demonstrated an acceptable entropy of 0.713.

The Black/African American (BAA)‐high harsh parenting/high neglect group (blue line, *n* = 611, 23.62%) was characterized by negative parent‐child interactions (i.e., early physical assault, psychological abuse, and neglect). The BAA‐low harsh parenting and neglect/low maternal depression group (gray line, *n* = 1713, 66.22%) had less negative parent‐child (i.e., early physical assault, psychological abuse, and neglect) and better peer interactions (i.e., high school connectedness and low peer bullying) and were more likely to be first‐generation immigrants. The BAA‐moderate harsh parenting/high neglect/chronic maternal depression group (orange line, *n* = 263, 10.17%) was characterized by chronic maternal depression and moderate to high negative parent‐child interaction, including physical assault and psychological abuse but with a particularly high level of neglect (Figure 1B).

#### White subsample

In the subsample of White children, the 4‐class solution was selected as the final model. While the 3‐class and 4‐class models were both competitive potential solutions with the same entropy of 0.74, the 4‐class model had a significant VLMR‐LRT (*p* = .039), which suggested that the 4‐class model was superior to the 3‐class model.

In the final model (Figure 1C), the White (W)‐first gen immigrant/low harsh parenting and neglect group (gray line, *n* = 410, 52.84%) was characterized by less negative parent‐child interactions, was more likely to be first‐generation immigrants, and had high levels of parent ethnic identity. The W‐low maternal education/high neighborhood safety group (blue line, *n* = 66, 8.51%) was the smallest group and was characterized by lower maternal education, better neighborhood safety, more school violence control measure, and more negative teacher attitude. This class also had the highest teacher‐rated neighborhood safety but the lowest neighborhood cohesion. The W‐high neglect/moderate harsh parenting group (orange line, *n* = 136, 17.53%) was characterized by high level of neglect and moderate level of physical assault and psychological abuse. The W‐high harsh parenting/high peer bullying group (yellow line, *n* = 164, 21.13%) was characterized by high level of physical assault, psychological abuse, and peer bullying.

### Aim 2: Examine predictive validity of early‐middle childhood environmental risk/resilience profiles for mental health outcomes in adolescence (age 15)

To summarize, across all race/ethnicity subsamples, environmental risk/resilience profiles entered in the first step of the stepwise model significantly predicted all mental health outcomes (Table 3). Post‐hoc comparisons using profile membership as predictor in regression models indicated that almost all environmental risk/resilience profile membership predicted both internalizing (anxiety, depression) and externalizing (attention deficit, oppositional defiant, conduct) psychopathology, although some profiles in the Black/African American and Hispanic/Latinx samples predicted externalizing only.

**TABLE 3:** Stepwise regression models with profile membership and irritability.

	Symptom outcomes at age 15	Depression		Anxiety		ADHD		ODD		CD	
	Predictors	*r*‐square change	*p*	*r*‐square change	*p*	*r*‐square change	*p*	*r*‐square change	*p*	*r*‐square change	*p*
Hispanic/Latinx	Profiles	0.010	0.02	0.001	0.7	0.032	<0.001	0.054	<0.001	0.037	<0.001
	Profiles + irritability	0.023	<0.001	0.026	<0.001	0.035	<0.001	0.051	<0.001	0.030	<0.001
Black/African American	Profiles	0.024	<0.001	0.012	<0.001	0.017	<0.001	0.035	<0.001	0.022	<0.001
	Profiles + irritability	0.026	<0.001	0.034	<0.001	0.031	<0.001	0.042	<0.001	0.028	<0.001
White	Profiles	0.031	0.002	0.015	0.072	0.033	<0.001	0.048	<0.001	0.041	<0.001
	Profiles + irritability	0.031	0.002	0.055	<0.001	0.020	0.002	0.029	<0.001	0.017	0.003

*Note*: The *p* values are adjusted for multiple comparison within each subsample using false discovery rate of 0.05. Profile membership was included in the initial model as the only predictor for psychopathology symptoms at age 15. Irritability at age 3 was added to the stepwise regression models as a predictor in addition to profile membership using the forward entry approach.

Abbreviations: ADHD, attention deficit hyperactivity disorder; CD, conduct disorder; ODD, oppositional defiant disorder.

Specifically, in the Black/African American subsample, post‐hoc comparisons indicated that BAA‐moderate harsh parenting/high neglect/chronic maternal depression group showed a higher level of symptoms in all five psychopathology categories (depression, anxiety, attention deficit, oppositional defiant, conduct symptoms) compared to BAA‐low harsh parenting and neglect/low maternal depression group, which showed a relatively low symptomatology in all categories. By contrast, the BAA‐high harsh parenting and neglect group was characterized by elevated externalizing (attention deficit, oppositional defiant, and conduct disorder) but not internalizing (depression, anxiety) symptoms.

In the Hispanic/Latinx sample, post‐hoc comparisons indicated that the HL‐school violence control/moderate harsh parenting group showed higher symptomatology across all psychopathology categories, as compared to the HL‐first generation immigrant/low harsh parenting group. The HL‐nonimmigrant/high harsh parenting/high bullying group showed elevation in externalizing (attention deficit, oppositional defiant, conduct) symptoms only.

In the White sample, post‐hoc analyses showed that, compared to the W‐first generation immigrant/low harsh parenting and neglect group, the W‐high neglect/moderate harsh parenting group and the W‐high harsh parenting/high peer bullying group both had significantly higher levels of symptomatology in all psychopathology categories but had an especially high level of conduct and oppositional defiant symptoms, respectively. The W‐low maternal education/high neighborhood safety group was characterized by elevation in externalizing (attention deficits, oppositional defiant, and conduct) symptoms. Analyses repeated with BCH approach, to account for class uncertainty in parameter estimation, showed consistent findings.

### Aim 3: Quantify the extent to which early childhood irritability (age 3) improves the prediction of adolescent mental health outcomes above and beyond environmental risk/resilience profiles

Adding child irritability at age 3 as a predictor, in addition to the environmental risk/resilience profiles, significantly improved model fit, explaining 1.7%–5.5% additional variance (Table 3 for *R* ^2^ change) in each psychopathology outcome, for all race/ethnicity groups. Higher levels of irritability predict increased symptomology in all psychopathology categories, above and beyond environmental risk/resilience profiles.

## DISCUSSION

Much research has been done in the past several decades to build the knowledge base of risk/resilience factors for psychopathology, yet wide gaps remain in our ability to implement such prediction in real‐world settings (Leibenluft & Kircanski, 2021). In order to match the growing public health need to identify at‐risk youths before vulnerable periods, such as adolescence, translational research with an eye toward integrating information across multiple levels is needed (Wakschlag et al., 2022). Here, we begin this translational process by not just comprehensively statistically synthesizing the risk/resilience literature but deriving the common risk/resilience patterns that youths may fall into. Capitalizing on a combination of theory‐guided multilevel risk/resilience factors with a data‐driven approach, this work contributes to the foundation for building toward multilevel flexible screening that integrates information from a variety of sources. Furthermore, the current study explored the additive utility of a promising, easily assessed child‐level trait, early childhood irritability, in improving prediction of mental health outcomes.

Some types of characteristics stood out by repeatedly driving differentiation among environmental risk/resilience subgroups across the racial/ethnic samples: immigration status, level of negative parent‐child interaction, and occurrences of maternal depression—all parent and family characteristics. By contrast, school and neighborhood characteristics distinguished subgroups to a lesser extent. That variations in patterns mainly driven by childhood experiences within the family context suggests that priority should be placed on family factors to improve the prediction of psychopathology in the development of risk calculation models, especially for settings where resources are limited (Of note, our findings do not imply that school and community factors are not important contexts for intervention, but rather that prediction is optimized by prioritizing parent and family level assessment at this developmental stage). It is critical to note that while our results highlight the relevance of family context in risk profiles, caregivers should not be expected to be the sole drivers for changes in the family environment. Instead, structural changes that support families, such as accessible and affordable healthcare, childcare, and family‐friendly policies, may have profound positive impact on the whole family. Future studies may consider exploring the efficacy of interventions that target the main risk factors across ecological levels, such as parent management training, school‐based therapy, and child tax credit. The current study examined the risk and resilience profiles for the race groups in parallel instead of comparatively, which allow us to preserve the nuances of environmental factors that are often tied to race. This approach allows us to examine the impact of differential experience on development with the consideration of the cultural implication linked to race with a caution to gear away from race‐based medicine. To preserve to power to detect racial/ethnic variation within the racial/ethnic groups (Celious & Oyserman, 2001; Whitfield et al., 2008), we leveraged the full sample for profile identification. Future studies may wish to focus on replicating the profiles in independent samples.

Our results moreover speak to the promise of early childhood irritability as a behavioral marker with additional predictive value over and above environmental risk/resilience profiles. As providers and researchers search for pragmatic, efficient ways to identify children at mental health risk, our results suggest that strategic assessment of a single dimension of early childhood behavior (irritability) plus selected family‐level environmental risk factors could be the best use of limited resources in clinics to screen and direct intervention resources. Put another way, early childhood screening could trigger multi‐level multi‐context assessment to account for child and environmental influences that modify risk and resilience pathways (Wakschlag et al., under review).

As expected, we identified a normative group based on risk and resilience factors for each racial/ethnic group, as well as 2–3 elevated patterns of risk factors. This is consistent with prior literature showing low plus elevated risk patterns. The elevated risk patterns however varied in how they predicted patterns of psychopathology. In our particular work, adolescents with elevated risk patterns either have broad‐spectrum psychopathology, that is both internalizing and externalizing, or externalizing patterns alone. It may be somewhat surprising that there was a not internalizing alone group. Yet of important note, parents reported on the adolescents' psychopathology and may not have had as much insight into the adolescents' internal state. Also of important note, in considering the full spectrum of internalizing and externalizing symptomatology, no group with elevated risk factors emerged as fully “resilient,” that is, each group has some psychopathology if not in the internalizing realm, then in the externalizing realm. This suggests that groups that have been labeled as resilient in prior research that only considered one aspect of psychopathology may actually experience detrimental effects in other aspects. Notably, we chose to only focus on internalizing and externalizing symptoms because of the high prevalence and relevance for developmental outcomes. Future studies may wish to explore the predictive values of environmental risk and resilience for other types of psychopathology such as psychosis.

While this study has several notable strengths, including a theory‐guided and data‐driven approach, large nation‐wide community sample, and longitudinal design spanning from birth to adolescence, there are several limitations. First, common in long‐spanning longitudinal studies, findings in this study may be affected by missing data due to attrition, despite that we used robust methods to address missingness. Second, while race/ethnicity is a significant component, we relied on parent‐reported racial identity; the child's own racial identity and acculturation are not included due to the lack of data availability and limited validity of child‐report at those ages. Third, this secondary data analysis was inherently constrained by the measure availability, and some measures had lower than ideal levels of reliability. However, we chose to leverage this large, nationwide sample to take advantage of the power to examine racial/ethnic subgroups and capitalize on the wide array of variables.

Ongoing work seeks to apply findings such as those presented here to the development of risk calculation algorithms that can reduce decision‐making burden on providers for intervention referral (Luby et al., 2019; MacNeill et al., 2021). To maximize flexibility and utility in real‐world situations, such algorithms need to output accurate recommendations with varied sets of information. By reducing the data complexity to a few common profiles that integrate information from multiple levels, this simplifies accurate clinical decision making for real world situations. In addition, our findings have the potential to improve the development of risk calculation algorithms through an “adaptive testing” type approach, where additional assessment is only needed when uncertainty for decision‐making is unacceptably high, because a child scores “on the edge” on a particular risk/resilience factor and/or it is unclear what risk/resilience profile they fit into. Here, our results begin the work of indicating the hierarchy of additional information needed to reduce uncertainty in these situations, as we found that some aspects were more critically predictive than others (e.g., child characteristics more so than neighborhood). Overall, this work can help to lay the foundation for a more efficient multi‐tiered information gathering process in mental health clinical settings to aid the decision making for determining intervention and prevention for children who may be at risk of developing psychological symptoms that can turn into entrenched psychiatric disorders in adulthood.

## AUTHOR CONTRIBUTIONS

**Qiongru Yu**: Conceptualization; data curation; formal analysis; investigation; methodology; visualization; writing – original draft; writing – review & editing. **Brianna Hernandez**: Conceptualization; data curation; formal analysis; project administration; writing – review & editing. **Conner Swineford**: Formal analysis; software; visualization. **Nia Walker**: Data curation; project administration; visualization. **Leigha MacNeill**: Conceptualization; methodology; writing – review & editing. **Yudong Zhang**: Conceptualization; methodology; writing – review & editing. **Lauren S. Wakschlag**: Conceptualization; methodology; writing – review & editing. **Jillian L. Wiggins**: Conceptualization; data curation; funding acquisition; investigation; methodology; project administration; supervision; writing – original draft; writing – review & editing.

## CONFLICT OF INTEREST STATEMENT

The authors have declared that they have no competing or potential conflicts of interest.

## ETHICAL CONSIDERATIONS

The data used in this study are de‐identified and therefore not considered human subject research.

## Supporting information

Supporting Information S1

## Data Availability

The data that support the findings of this study are publicly available upon request from the Future of Families & Child Wellbeing Study at https://ffcws.princeton.edu.

## References

[jcv212180-bib-0001] Aalto‐Setälä, T. , Marttunen, M. , Tuulio‐Henriksson, A. , Poikolainen, K. , & Lönnqvist, J. (2002). Depressive symptoms in adolescence as predictors of early adulthood depressive disorders and maladjustment. American Journal of Psychiatry, 159(7), 1235–1237. 10.1176/appi.ajp.159.7.1235 12091207

[jcv212180-bib-0002] Achenbach, T. M. (1992). Manual for the child behavior checklist/2‐3 and 1992 profile. Dept. of Psychiatry, University of Vermont.

[jcv212180-bib-0003] Achenbach, T. M. , & Rescorla, L. (2001). Manual for the ASEBA school‐age forms & profiles: An integrated system of multi‐informant assessment.

[jcv212180-bib-0004] Arrington, E. G. , & Wilson, M. N. (2000). A re‐examination of risk and resilience during adolescence: Incorporating culture and diversity. Journal of Child and Family Studies, 9(2), 221–230. 10.1023/A:1009423106045

[jcv212180-bib-0005] Asparouhov, T. , & Muthén, B. (2014). Auxiliary variables in mixture modeling: Using the BCH method in Mplus to estimate a distal outcome model and an arbitrary secondary model. Mplus Web Notes, 21(2), 1–22.

[jcv212180-bib-0006] Bailey, Z. D. , Krieger, N. , Agénor, M. , Graves, J. , Linos, N. , & Bassett, M. T. (2017). Structural racism and health inequities in the USA: Evidence and interventions. The Lancet, 389(10077), 1453–1463. 10.1016/S0140-6736(17)30569-X 28402827

[jcv212180-bib-0007] Bronfenbrenner, U. (1977). Toward an experimental ecology of human development. American Psychologist, 32(7), 513–531. 10.1037/0003-066X.32.7.513

[jcv212180-bib-0008] Burstein, M. , Ginsburg, G. S. , Petras, H. , & Ialongo, N. (2010). Parent psychopathology and youth internalizing symptoms in an urban community: A latent growth model analysis. Child Psychiatry and Human Development, 41(1), 61–87. 10.1007/s10578-009-0152-y 19669407 PMC3373962

[jcv212180-bib-0009] Celious, A. , & Oyserman, D. (2001). Race from the inside: An emerging heterogeneous race model. Journal of Social Issues, 57(1), 149–165. 10.1111/0022-4537.00206

[jcv212180-bib-0010] Copeland‐Linder, N. , Lambert, S. F. , & Ialongo, N. S. (2010). Community violence, protective factors, and adolescent mental health: A profile analysis. Journal of Clinical Child and Adolescent Psychology, 39(2), 176–186. 10.1080/15374410903532601 20390809 PMC3584688

[jcv212180-bib-0011] Dunn, V. J. , Abbott, R. A. , Croudace, T. J. , Wilkinson, P. , Jones, P. B. , Herbert, J. , & Goodyer, I. M. (2011). Profiles of family‐focused adverse experiences through childhood and early adolescence: The ROOTS project a community investigation of adolescent mental health. BMC Psychiatry, 11(1), 109. 10.1186/1471-244X-11-109 21736727 PMC3199756

[jcv212180-bib-0012] Green, J. G. , McLaughlin, K. A. , Berglund, P. A. , Gruber, M. J. , Sampson, N. A. , Zaslavsky, A. M. , & Kessler, R. C. (2010). Childhood adversities and adult psychiatric disorders in the national comorbidity survey replication I: Associations with first onset of DSM‐IV disorders. Archives of General Psychiatry, 67(2), 113–123. 10.1001/archgenpsychiatry.2009.186 20124111 PMC2822662

[jcv212180-bib-0013] Harnett, N. G. (2020). Neurobiological consequences of racial disparities and environmental risks: A critical gap in understanding psychiatric disorders. Neuropsychopharmacology, 45(8), 1247–1250. 10.1038/s41386-020-0681-4 32330926 PMC7411049

[jcv212180-bib-0014] Heleniak, C. , Jenness, J. L. , Vander Stoep, A. , McCauley, E. , & McLaughlin, K. A. (2016). Childhood maltreatment exposure and disruptions in emotion regulation: A transdiagnostic pathway to adolescent internalizing and externalizing psychopathology. Cognitive Therapy and Research, 40(3), 394–415. 10.1007/s10608-015-9735-z 27695145 PMC5042349

[jcv212180-bib-0015] Kuckertz, J. M. , Mitchell, C. , & Wiggins, J. L. (2018). Parenting mediates the impact of maternal depression on child internalizing symptoms. Depression and Anxiety, 35(1), 89–97. 10.1002/da.22688 28962070 PMC5760303

[jcv212180-bib-0016] Lazarevic, V. , Toledo, G. , & Wiggins, J. L. (2020). Influence of maternal ethnic–racial identity on children’s internalizing symptom trajectories. Journal of Experimental Psychopathology, 11(1), 2043808719898024. 10.1177/2043808719898024

[jcv212180-bib-0017] Leibenluft, E. , & Kircanski, K. (2021). Chronic irritability in youth: A reprise on challenges and opportunities toward meeting unmet clinical needs. Child and Adolescent Psychiatric Clinics of North America, 30(3), 667–683. 10.1016/j.chc.2021.04.014 34053693 PMC13317484

[jcv212180-bib-0018] Luby, J. , Allen, N. , Estabrook, R. , Pine, D. S. , Rogers, C. , Krogh‐Jespersen, S. , Norton, E. S. , & Wakschlag, L. (2019). Mapping infant neurodevelopmental precursors of mental disorders: How synthetic cohorts & computational approaches can be used to enhance prediction of early childhood psychopathology. Behaviour Research and Therapy, 123, 103484. 10.1016/j.brat.2019.103484 31734549 PMC7667707

[jcv212180-bib-0019] MacNeill, L. A. , Allen, N. B. , Poleon, R. B. , Vargas, T. , Osborne, K. J. , Damme, K. S. F. , Barch, D. M. , Krogh‐Jespersen, S. , Nielsen, A. N. , Norton, E. S. , Smyser, C. D. , Rogers, C. E. , Luby, J. L. , Mittal, V. A. , & Wakschlag, L. S. (2021). Translating RDoC to real‐world impact in developmental psychopathology: A neurodevelopmental framework for application of mental health risk calculators. Development and Psychopathology, 33(5), 1665–1684. 10.1017/S0954579421000651 35095215 PMC8794223

[jcv212180-bib-0020] Mah, V. K. , & Ford‐Jones, E. L. (2012). Spotlight on middle childhood: Rejuvenating the ‘forgotten years’. Paediatrics and Child Health, 17(2), 81–83. 10.1093/pch/17.2.81 23372398 PMC3299351

[jcv212180-bib-0021] McCartney, K. , Burchinal, M. R. , & Kristen, L. B. (2006). Best practices in quantitative methods for developmentalists. Monographs of the Society for Research in Child Development, 71(3), i–145. http://www.jstor.org/stable/4121953 10.1111/j.1540-5834.2006.07103001.x 17199773

[jcv212180-bib-0022] McLaughlin, K. A. , Garrad, M. C. , & Somerville, L. H. (2015). What develops during emotional development? A component process approach to identifying sources of psychopathology risk in adolescence. Dialogues in Clinical Neuroscience, 17(4), 403–410. 10.31887/DCNS.2015.17.4/kmclaughlin 26869841 PMC4734878

[jcv212180-bib-0023] Merikangas, K. R. , He, J.‐p. , Burstein, M. , Swanson, S. A. , Avenevoli, S. , Cui, L. , Benjet, C. , Georgiades, K. , & Swendsen, J. (2010). Lifetime prevalence of mental disorders in U.S. adolescents: Results from the national comorbidity survey replication–adolescent supplement (NCS‐A). Journal of the American Academy of Child & Adolescent Psychiatry, 49(10), 980–989. 10.1016/j.jaac.2010.05.017 20855043 PMC2946114

[jcv212180-bib-0024] Mittal, V. A. , & Wakschlag, L. S. (2017). Research domain criteria (RDoC) grows up: Strengthening neurodevelopment investigation within the RDoC framework. Journal of Affective Disorders, 216, 30–35. 10.1016/j.jad.2016.12.011 28010957 PMC5471127

[jcv212180-bib-0025] Moylan, C. A. , Herrenkohl, T. I. , Sousa, C. , Tajima, E. A. , Herrenkohl, R. C. , & Russo, M. J. (2010). The effects of child abuse and exposure to domestic violence on adolescent internalizing and externalizing behavior problems. Journal of Family Violence, 25(1), 53–63. 10.1007/s10896-009-9269-9 20495613 PMC2872483

[jcv212180-bib-0026] Paat, Y.‐F. (2013). Working with immigrant children and their families: An application of Bronfenbrenner's ecological systems theory. Journal of Human Behavior in the Social Environment, 23(8), 954–966. 10.1080/10911359.2013.800007

[jcv212180-bib-0027] Persell, C. H. (1981). Genetic and cultural deficit theories: Two sides of the same racist coin. Journal of Black Studies, 12(1), 19–37. 10.1177/002193478101200102

[jcv212180-bib-0028] Quinlan, E. B. , Barker, E. D. , Luo, Q. , Banaschewski, T. , Bokde, A. L. W. , Bromberg, U. , Büchel, C. , Desrivières, S. , Flor, H. , Frouin, V. , Garavan, H. , Chaarani, B. , Gowland, P. , Heinz, A. , Brühl, R. , Martinot, J.‐L. , Martinot, M.‐L. P. , Nees, F. , Orfanos, D. P. , … Consortium, I. (2020). Peer victimization and its impact on adolescent brain development and psychopathology. Molecular Psychiatry, 25(11), 3066–3076. 10.1038/s41380-018-0297-9 30542059

[jcv212180-bib-0029] Reichman, N. E. , Teitler, J. O. , Garfinkel, I. , & McLanahan, S. S. (2001). Fragile families: Sample and design. Children and Youth Services Review, 23(4–5), 303–326. 10.1016/s0190-7409(01)00141-4

[jcv212180-bib-0030] Reijntjes, A. , Kamphuis, J. H. , Prinzie, P. , & Telch, M. J. (2010). Peer victimization and internalizing problems in children: A meta‐analysis of longitudinal studies. Child Abuse & Neglect, 34(4), 244–252. 10.1016/j.chiabu.2009.07.009 20304490

[jcv212180-bib-0031] Sampson, R. J. , Raudenbush, S. W. , & Earls, F. (1997). Neighborhoods and violent crime: A multilevel study of collective efficacy. Science, 277(5328), 918–924. 10.1126/science.277.5328.918 9252316

[jcv212180-bib-0032] Shochet, I. M. , Dadds, M. R. , Ham, D. , & Montague, R. (2006). School connectedness is an underemphasized parameter in adolescent mental health: Results of a community prediction study. Journal of Clinical Child and Adolescent Psychology, 35(2), 170–179. 10.1207/s15374424jccp3502_1 16597213

[jcv212180-bib-0033] Takizawa, R. , Maughan, B. , & Arseneault, L. (2014). Adult health outcomes of childhood bullying victimization: Evidence from a five‐decade longitudinal British birth cohort. American Journal of Psychiatry, 171(7), 777–784. 10.1176/appi.ajp.2014.13101401 24743774

[jcv212180-bib-0034] Wakschlag, L. S. , Davis, M. M. , & Smith, J. D. (under review). Pediatric irritability as actionable transdiagnostic marker of early mental health risk.

[jcv212180-bib-0035] Wakschlag, L. S. , Finlay‐Jones, A. L. , MacNeill, L. A. , Kaat, A. J. , Brown, C. H. , Davis, M. M. , Franklin, P. , Berkel, C. , Krogh‐Jespersen, S. , & Smith, J. D. (2022). Don't get lost in translation: Integrating developmental and implementation sciences to accelerate real‐world impact on children's development, health, and wellbeing. Frontiers in Public Health, 10. 10.3389/fpubh.2022.827412 PMC9046665 35493380

[jcv212180-bib-0036] Wakschlag, L. S. , Perlman, S. B. , Blair, R. J. , Leibenluft, E. , Briggs‐Gowan, M. J. , & Pine, D. S. (2018). The neurodevelopmental basis of early childhood disruptive behavior: Irritable and callous phenotypes as exemplars. American Journal of Psychiatry, 175(2), 114–130. 10.1176/appi.ajp.2017.17010045 29145753 PMC6075952

[jcv212180-bib-0037] Wakschlag , L.S., Pool, M.N. , Adam, B. , Norton, K.J. , Rogers, A. , Smyser, L. , & Allen, H. (under review). Predictive utility of irritability “in context”: Proof‐of‐principle for an early childhood mental health risk calculator. 10.1080/15374416.2023.2188553 PMC10533737 36975800

[jcv212180-bib-0038] Whitfield, K. E. , Allaire, J. C. , Belue, R. , & Edwards, C. L. (2008). Are comparisons the answer to understanding behavioral aspects of aging in racial and ethnic groups? The Journals of Gerontology: Series B, 63(5), P301–P308. 10.1093/geronb/63.5.P301 PMC3663459 18818445

[jcv212180-bib-0039] Wiggins, J. L. , Briggs‐Gowan, M. J. , Estabrook, R. , Brotman, M. A. , Pine, D. S. , Leibenluft, E. , & Wakschlag, L. S. (2018). Identifying clinically significant irritability in early childhood. Journal of the American Academy of Child & Adolescent Psychiatry, 57(3), 191–199.e192. 10.1016/j.jaac.2017.12.008 29496128 PMC5860673

[jcv212180-bib-0040] Wiggins, J. L. , Mitchell, C. , Hyde, L. W. , & Monk, C. S. (2015). Identifying early pathways of risk and resilience: The codevelopment of internalizing and externalizing symptoms and the role of harsh parenting. Development and Psychopathology, 27(4 Pt 1), 1295–1312. 10.1017/S0954579414001412 26439075 PMC4961476

[jcv212180-bib-0041] Wiggins, J. L. , Mitchell, C. , Stringaris, A. , & Leibenluft, E. (2014). Developmental trajectories of irritability and bidirectional associations with maternal depression. Journal of the American Academy of Child & Adolescent Psychiatry, 53(11), 1191–1205.e1194. 10.1016/j.jaac.2014.08.005 25440309 PMC4254549

[jcv212180-bib-0042] Wiggins, J. L. , Rosario, A. U. , MacNeill, L. A. , Krogh‐Jespersen, S. , Smith, J. D. , & Wakschlag, L. S. (under review). The clinically optimized irritability score: Pragmatic early assessment of mental disorder risk. 10.1002/mpr.1991 PMC10654826 37728118

[jcv212180-bib-0043] Wright, J. L. , Davis, W. S. , Joseph, M. M. , Ellison, A. M. , Heard‐Garris, N. J. , & Johnson, T. L. (2022). Eliminating race‐based medicine. Pediatrics, 150(1). 10.1542/peds.2022-057998 35491483

[jcv212180-bib-0044] Zeiders, K. H. , Roosa, M. W. , Knight, G. P. , & Gonzales, N. A. (2013). Mexican American Adolescents' profiles of risk and mental health: A person‐centered longitudinal approach. Journal of Adolescence, 36(3), 603–612. 10.1016/j.adolescence.2013.03.014 23608782 PMC3743430

