# Supplementary material for: Toward an optimized assessment of adolescent psychopathology risk: Multilevel environmental profiles and child irritability as predictors
Source: JCPP Adv. 2023 Jun 13;3(4):e12180. doi: 10.1002/jcv2.12180 (PMC10694535; doi:10.1002/jcv2.12180)
Supplement: Supplementary file 1 — Supporting Information S1 [file JCV2-3-e12180-s001.docx]

Toward an optimized assessment of adolescent psychopathology risk:

Multilevel environmental profiles and child irritability as predictors

**Supporting Information**

Table S1. Latent profile analysis model results for the Hispanic subsample

|  | HL-first gen immigrant/low harsh parenting and neglect group | | HL-nonimmigrant/high harsh parenting/high bullying group | | HL-school violence control/moderate harsh parenting group | |
| --- | --- | --- | --- | --- | --- | --- |
|  | Est. (S.E.) | *P* value | Est. (S.E.) | *P* value | Est. (S.E.) | *P* value |
| Early physical assault | 0.83 (0.026) | <.0001 | 1.743 (0.074) | <.0001 | 1.33 (0.104) | <.0001 |
| Change in physical assault | -0.066 (0.002) | <.0001 | -0.14 (0.01) | <.0001 | 0.033 (0.017) | 0.055 |
| Early psychological abuse | 1.297 (0.031) | <.0001 | 2.095 (0.054) | <.0001 | 1.851 (0.076) | <.0001 |
| Change in psychological abuse | -0.038 (0.002) | <.0001 | -0.054 (0.007) | <.0001 | 0.089 (0.015) | <.0001 |
| Neglect | 0.247 (0.019) | <.0001 | 0.654 (0.072) | <.0001 | 0.739 (0.122) | <.0001 |
| Maternal education | 0.783 (0.03) | <.0001 | 0.916 (0.062) | <.0001 | 0.821 (0.17) | <.0001 |
| Neighborhood cohesion | 2.174 (0.032) | <.0001 | 2.091 (0.06) | <.0001 | 2.031 (0.14) | <.0001 |
| Neighborhood safety | 0.695 (0.033) | <.0001 | 0.632 (0.061) | <.0001 | 0.829 (0.139) | <.0001 |
| Parental cultural identity | 2.12 (0.024) | <.0001 | 2.072 (0.045) | <.0001 | 1.963 (0.1) | <.0001 |
| Peer bullying | 0.478 (0.029) | <.0001 | 0.677 (0.068) | <.0001 | 0.527 (0.105) | <.0001 |
| School connectedness | 3.165 (0.039) | <.0001 | 3.055 (0.076) | <.0001 | 2.872 (0.141) | <.0001 |
| Teacher attitude | 3.034 (0.032) | <.0001 | 3.02 (0.064) | <.0001 | 3.079 (0.099) | <.0001 |
| School violence control | 0.503 (0.01) | <.0001 | 0.474 (0.019) | <.0001 | 0.581 (0.035) | <.0001 |
| Second gen immigrant | 1.885 (0.125) | <.0001 | 1.538 (0.222) | <.0001 | 1.461 (0.437) | 0.001 |
| First gen immigrant | -0.2 (0.089) | 0.025 | 0.698 (0.182) | <.0001 | 0.383 (0.405) | 0.345 |
| Maternal depression - once | 0.922 (0.079) | <.0001 | -0.019 (0.142) | 0.891 | 0.081 (0.263) | 0.759 |
| Maternal depression - chronic | 2.23 (0.12) | <.0001 | 1.303 (0.161) | <.0001 | 1.332 (0.333) | <.0001 |

Table S2. Latent profile analysis model results for the Black/African American subsample

|  | BAA-high harsh parenting/high neglect group | | BAA-low harsh parenting and neglect/low maternal depression group | | BAA-moderate harsh parenting/high neglect/chronic maternal depression group | |
| --- | --- | --- | --- | --- | --- | --- |
|  | Est. (S.E.) | *P* value | Est. (S.E.) | *P* value | Est. (S.E.) | *P* value |
| Early physical assault | 2.015 (0.056) | <.0001 | 0.999 (0.028) | <.0001 | 1.803 (0.08) | <.0001 |
| Change in physical assault | -0.146 (0.009) | <.0001 | -0.067 (0.002) | <.0001 | -0.085 (0.01) | <.0001 |
| Early psychological abuse | 2.227 (0.037) | <.0001 | 1.425 (0.029) | <.0001 | 2.151 (0.055) | <.0001 |
| Change in psychological abuse | -0.05 (0.007) | <.0001 | -0.031 (0.002) | <.0001 | 0.009 (0.009) | .326 |
| Neglect | 0.226 (0.079) | .004 | 0.168 (0.025) | <.0001 | 1.571 (0.221) | <.0001 |
| Maternal education | 1.046 (0.049) | <.0001 | 1.048 (0.027) | <.0001 | 0.902 (0.064) | <.0001 |
| Neighborhood cohesion | 2.144 (0.047) | <.0001 | 2.209 (0.027) | <.0001 | 1.995 (0.075) | <.0001 |
| Neighborhood safety | 0.916 (0.053) | <.0001 | 0.847 (0.029) | <.0001 | 0.878 (0.083) | <.0001 |
| Parental cultural identity | 2.038 (0.034) | <.0001 | 2.007 (0.023) | <.0001 | 2.047 (0.053) | <.0001 |
| Peer bullying | 0.684 (0.045) | <.0001 | 0.647 (0.028) | <.0001 | 0.808 (0.075) | <.0001 |
| School connectedness | 3.062 (0.056) | <.0001 | 3.084 (0.034) | <.0001 | 2.958 (0.08) | <.0001 |
| Teacher attitude | 2.894 (0.051) | <.0001 | 2.906 (0.028) | <.0001 | 2.852 (0.077) | <.0001 |
| School violence control | 0.588 (0.014) | <.0001 | 0.579 (0.008) | <.0001 | 0.563 (0.02) | <.0001 |
| Second gen immigrant | 2.396 (0.213) | <.0001 | 2.384 (0.129) | <.0001 | 2.64 (0.436) | <.0001 |
| First gen immigrant | 2.406 (0.232) | <.0001 | 1.986 (0.115) | <.0001 | 2.996 (0.492) | <.0001 |
| Maternal depression - once | 0.218 (0.13) | .094 | 0.712 (0.065) | <.0001 | -0.436 (0.16) | .006 |
| Maternal depression - chronic | 1.493 (0.141) | <.0001 | 1.847 (0.088) | <.0001 | 0.468 (0.222) | 0.035 |

Table S3. Latent profile analysis model results for the white subsample

|  | W-low maternal education/high neighborhood safety group | | W-first gen immigrant/low harsh parenting and neglect group | | W-high neglect/moderate harsh parenting group | | W-high harsh parenting/high peer bullying group | |
| --- | --- | --- | --- | --- | --- | --- | --- | --- |
|  | Est. (S.E.) | *P* value | Est. (S.E.) | *P* value | Est. (S.E.) | *P* value | Est. (S.E.) | *P* value |
| Early physical assault | 1.247 (0.089) | <.0001 | 0.782 (0.039) | <.0001 | 1.177 (0.051) | <.0001 | 1.58 (0.091) | <.0001 |
| Change in physical assault | -0.081 (0.012) | <.0001 | -0.066 (0.003) | <.0001 | -0.093 (0.005) | <.0001 | -0.13 (0.009) | <.0001 |
| Early psychological abuse | 1.675 (0.091) | <.0001 | 1.268 (0.041) | <.0001 | 1.729 (0.053) | <.0001 | 1.967 (0.071) | <.0001 |
| Change in psychological abuse | -0.024 (0.011) | .026 | -0.028 (0.003) | <.0001 | -0.03 (0.007) | <.0001 | -0.048 (0.008) | <.0001 |
| Neglect | 0 (0) | .748 | 0 (0) | .702 | 1.194 (0.034) | <.0001 | 0 (0) | .713 |
| Maternal education | 1.086 (0.136) | <.0001 | 2.046 (0.074) | <.0001 | 1.976 (0.093) | <.0001 | 1.53 (0.103) | <.0001 |
| Neighborhood cohesion | 2.192 (0.122) | <.0001 | 2.585 (0.042) | <.0001 | 2.513 (0.052) | <.0001 | 2.46 (0.065) | <.0001 |
| Neighborhood safety | 1.215 (0.096) | <.0001 | 0.086 (0.016) | <.0001 | 0.192 (0.039) | <.0001 | 0.101 (0.028) | <.0001 |
| Parental cultural identity | 1.518 (0.115) | <.0001 | 1.874 (0.047) | <.0001 | 1.742 (0.061) | <.0001 | 1.538 (0.068) | <.0001 |
| Peer bullying | 0.499 (0.109) | <.0001 | 0.453 (0.052) | <.0001 | 0.483 (0.055) | <.0001 | 0.823 (0.133) | <.0001 |
| School connectedness | 3.26 (0.166) | <.0001 | 3.259 (0.068) | <.0001 | 2.938 (0.087) | <.0001 | 2.931 (0.155) | <.0001 |
| Classroom climate | 2.802 (0.113) | <.0001 | 3.272 (0.043) | <.0001 | 3.167 (0.07) | <.0001 | 3.309 (0.07) | <.0001 |
| School violence control | 0.57 (0.027) | <.0001 | 0.43 (0.015) | <.0001 | 0.466 (0.018) | <.0001 | 0.444 (0.022) | <.0001 |
| Binary variable threshold (logit) |  |  |  |  |  |  |  |  |
| Second generation immigrant | 2.099 (0.503) | 999 | 2.601 (0.256) | <.0001 | 2.011 (0.285) | <.0001 | 2.544 (0.426) | <.0001 |
| First generation immigrant | 15 (0) | 0.337 | 1.945 (0.203) | <.0001 | 2.731 (0.418) | <.0001 | 3.072 (0.596) | <.0001 |
| Maternal depression - once | 0.293 (0.305) | 0.002 | 1.258 (0.186) | <.0001 | -0.083 (0.174) | 0.633 | 0.258 (0.195) | .185 |
| Maternal depression - chronic | 1.134 (0.37) | 999 | 2.752 (0.314) | <.0001 | 0.854 (0.185) | <.0001 | 1.436 (0.257) | <.0001 |

Note: estimates and standard error are in the original scale of the measures. Immigration status and maternal depression variables were binary, and the estimates for these variables represent binary logit thresholds.
